# Microsegregation Model Including Convection and Tip Undercooling: Application to Directional Solidification and Welding

**DOI:** 10.3390/ma11071252

**Published:** 2018-07-20

**Authors:** Thomas Billotte, Dominique Daloz, Bernard Rouat, Guillaume Tirand, Jacob R. Kennedy, Vincent Robin, Julien Zollinger

**Affiliations:** 1 Department of Metallurgy and Materials Science and Engineering, Institut Jean Lamour, UMR CNRS 7198, Université de Lorraine, 54000 Nancy, France; t.billotte@isgroupe.com (T.B.); bernard.rouat@univ-lorraine.fr (B.R.); jacob-roman.kennedy@univ-lorraine.fr (J.R.K.); julien.zollinger@univ-lorraine.fr (J.Z.); 2 Laboratory of Excellence on Design of Alloy Metals for Low-mAss Structures (‘LabEx DAMAS’), Université de Lorraine, 57073 Metz, France; 3 AREVA, Technical Center, 71100 Saint-Marcel, France; guillaume.tirand@framatome.com; 4 AREVA, Engineering & Projects, 69006 Lyon, France; vincent.robin@edf.fr

**Keywords:** microsegregation, gas tungsten arc welding, directional solidification, FM52 filler metal, ERNiCrFe-7, tip undercooling

## Abstract

The microsegregation behavior of alloy filler metal 52 (FM 52) was studied using microprobe analysis on two different solidification processes. First, microsegregation was characterized in samples manufactured by directional solidification, and then by gas tungsten arc welding (GTAW). The experimental results were compared with Thermo-Calc calculations to verify their accuracy. It was confirmed that the thermodynamic database predicts most alloying elements well. Once this data had been determined, several tip undercooling calculations were carried out for different solidification conditions in terms of fluid flow and thermal gradient values. These calculations allowed the authors to develop a parametrization card for the constants of the microsegregation model, according to the process parameters (e.g., convection in melt pool, thermal gradient, and growth velocity). A new model of microsegregation, including convection and tip undercooling, is also proposed. The Tong–Beckermann microsegregation model was used individually and coupled with a modified Kurz-Giovanola-Trivedi (KGT) tip undercooling model, in order to take into account the convection in the fluid flow at the dendrite tip. Model predictions were compared to experimental results and showed the microsegregation evolution accurately.

## 1. Introduction

The first pressurized water reactor (PWR) components were manufactured from stainless steel and nickel A600 alloys. Welding of the reactors was performed using FM82 filler metal in order to maintain both mechanical and anti-corrosion properties. Since 1990, A690 nickel alloy has replaced the A600 alloy in order to improve stress corrosion cracking resistance [1]. In addition, a new filler metal, FM52, was developed. A690 alloy mainly consists of an austenitic matrix containing M_23_C_6_ (MC) carbides [2,3,4]. MC carbides are also observed in the filler metal FM52 [5,6,7], as well as some titanium nitrides, as reported in the literature [8,9].

A690 and FM52 satisfy the specification for corrosion resistance [10], but suffer from a tendency for ductility dip cracking (DDC) during welding [11,12,13,14,15,16,17]. Different factors may affect the DDC sensitivity, such as alloy composition, element segregation (phosphorous and sulfur), secondary phase precipitation at grain boundaries, grain boundary sliding, or grain boundary orientation [12]. Since 2000, this has led to many developments to improve the DCC resistance, focusing mainly on the chemical composition [18]. FM52 can now be reinforced using boron and zirconium for FM52M [19], and using molybdenum and niobium for FM52MSS, to improve its mechanical properties [20,21]. However, segregation of boron at the grain boundaries promotes the formation of low melting point compounds, generating liquation cracks [22]. This cracking behavior can be further exacerbated by the presence of oxide bifilms [23]; the same effect is noted for zirconium [24]. The importance of back-diffusion to reduce solidification cracking has also recently been shown [25], along with the fact that microsegregation in gas tungsten arc welding (GTAW) can be somewhat controlled by the use of pulsed current [26]. It is critical then, for development and application, to thoroughly understand how segregation occurs and how to model it. It is useful to simulate solidification structures using a model in which a cellular automaton is coupled with a finite element mesh (CAFE) [27,28]. 

When such predictive modeling tools are applied to welding or additive manufacturing, the Scheil rule (or truncated Scheil [29])—based on the hypothesis that no solid diffusion occurs—is employed, due to the relatively rapid solidification compared to other casting processes [30]. Time allowed for back-diffusion in the solid is low in rapid solidification, which correlates well with the Scheil model hypothesis. However, the structures formed are much finer than those obtained with slower solidification processes. This means that less time is needed for elements to back-diffuse into the solid and, thus, the applicability of the Scheil rule becomes more problematic [31]. Additionally, the intense convection that occurs in the weld pool may affect the solutal build-up associated with solidification, and thereby modify the dendrite tip undercooling. This is not taken into account in the Scheil description, and may affect the initial conditions for microsegregation.

Currently, there is no published work which specifically regards dendrite tip undercooling and convection in the melt pool [32]. Convection is in the order of tens of centimeters per second during welding. Thus, it is relevant to quantify the convection effect on undercooling and include it in a microsegregation model. The ability of the Scheil model to correctly represent the microsegregation in such rapid growth conditions may also be investigated. This paper will address both of these points.

In this work, quenched directional solidification (QDS) and gas tungsten arc welding (GTAW) of FM52 were carried out. From the experiments, the microsegregation was characterized. A KGT [33] tip undercooling model—which took fluid flow into account [34]—was applied, in order to quantify the importance of convection on the undercooling. The Tong–Beckermann [35] microsegregation model was also used, which could account for the importance of back-diffusion. This model, together with the classical lever rule and Gulliver–Scheil model, are compared with the experimental results. These results are discussed, and rules for better use of microsegregation models are proposed. 

## 2. Experimental Section

The FM52 alloy was provided by AREVA. The chemical composition of the alloy was measured using the electron probe micro-analysis (EPMA) on a JEOL JXA 8530F apparatus (JEOL, Tokyo, Japan). Table 1 shows the results of this analysis, compared with the manufacturer’s specifications.

The solidus and liquidus temperatures were determined using a SETARAM Setsys 16/18 differential thermal analysis (DTA) (SETARAM, Lyon, France). Four cooling and heating rates were applied, ranging from 2 to 20 K/min. The results were examined using the Boettinger [37] recommendations and the Bobadilla observations [38]. The DTA results for solidus and liquidus temperatures are summarized in Table 2, and compared with thermodynamic calculations performed using the Thermo-Calc software (Thermo-Calc 2018b, Thermo-Calc software, Solna, Sweden) with the TTNi8 database [39]. The solidification range (ΔT_0_) found by DTA analysis was 10.6 °C, which is very close to the value (13 °C) found by Wu and Tsai [40]. As seen in Table 2, the values given by the TTNi8 database for the FM52 alloy (considering all alloying elements) does not reflect the experimental DTA measurements, especially for the solidification range (36 °C vs. 10.6 °C). If only the major constituent elements of the alloy are considered (Cr, Fe), the calculated solidification range is 8.4 °C, showing much better agreement with the DTA measurements.

Experiments involving quenching during directional solidification (QDS) were performed with a specially designed Bridgman type furnace. This method allows the thermal gradient (*G*) and the solidification velocity (*V*) to be controlled independently. The quench imposes a cooling rate of 100 °C/s, and is able to freeze the microstructure and microsegregations during solidification [41,42]. Alloy FM52 was melted and cast into cylindrical rods, 5 mm in diameter, which were used in the Bridgman furnace. After the QDS experiments, transverse cross-sections were cut along the length of the mushy zone. Each section corresponds to a given temperature before the quench and, for a given solid fraction, the morphology and the amount of segregation in the solidifying alloy. 

The welding experiments were performed using gas tungsten arc welding (GTAW) under argon. The filler metal was FM52 alloy and the welding plates were A690 nickel alloy. Contrary to the QDS experiments, the solidification operating parameters are not controlled in the GTAW process. For this study, the growth velocity was considered equal to the welding speed, and the thermal gradient was determined using the SYSWELD software (2017, ESI, Paris, France). The solidification parameters, and *G* and *V* for the QDS and welding experiments are given in Table 3.

To characterize microsegregation, EPMA measurements were performed using regular analysis grids [41,42,43,44]. The parameters for the systematic sampling were determined using a previously developed method [44]. The dimensions of the analysis grids were the same for all the analyzed samples, e.g., 196 measurements with a 100 μm step size, so that the sampling parameter, *r*, defined by $r=\frac{step}{\lambda_{1}}$, was always less than or equal to 10 [44].

The sorting strategy used was based on the element which was known to have the greatest tendency to segregate in the liquid [41]. In this work, titanium was selected, as Ti is known to segregate in the liquid during solidification [9]. All other elements were arranged following the Ti values in ascending order. In order to compare them, the microsegregation profiles were normalized to the mean composition calculated from all 196 points.

## 3. Microstructure and Microsegregation

### 3.1. Secondary Dendrite Arm Spacing (SDAS) Law

The scale of microsegregation was found using secondary dendrite arm spacing (SDAS) measurements. It was necessary to know the SDAS evolution, with respect to the solidification parameters, in order to utilize the data in the Tong–Beckermann (TB) model. For this reason, several QDS experiments were performed with different solidification conditions. For each experiment, several micrographs from the longitudinal section of the QDS samples were taken to measure the SDAS. This was also carried out for the GTAW experiments. 

**Figure 1:**
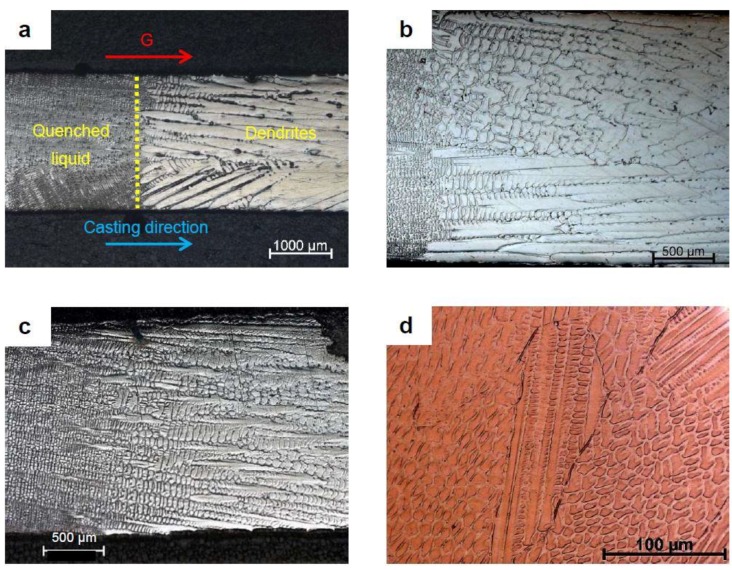
Micrographs for the secondary dendrite arm spacing (SDAS) measurement after electrolytic etching, using a 10 g H_2_C_2_O_4_ with 100 mL H_2_O reagent, under the applied potential 4 V DC for 30–40 s: (**a**) QDS for a solidification velocity *V* = 0.013 mm/s and thermal gradient (*G*) = 3000 K/m; (**b**) QDS for *V* = 0.03 mm/s and *G* = 5300 K/m; (**c**) QDS for *V* = 0.09 mm/s and G = 5300 K/m; and (**d**) gas tungsten arc welding (GTAW).

Figure 1 illustrates the different sizes of the solidification structures from the experiments. As shown in Figure 1a, the solidification front is clearly visible on the QDS micrographs (Figure 1a–c). The dendrites grow following the thermal gradient, however, in the case of GTAW, it was less clear, since the thermal gradient vector was not constant. Nevertheless, a dendritic structure can still be observed in Figure 1d. The evolution of the size of the solidification structures is also visible in Figure 1. As the product of *G* and *V* increased, the size of the structures decreased, as can be seen by comparing the QDS experiments. This is even more evident with the GTAW experiments, where the cooling rate is 1000 times higher than the maximum cooling rate obtained with QDS.

**Figure 2:**
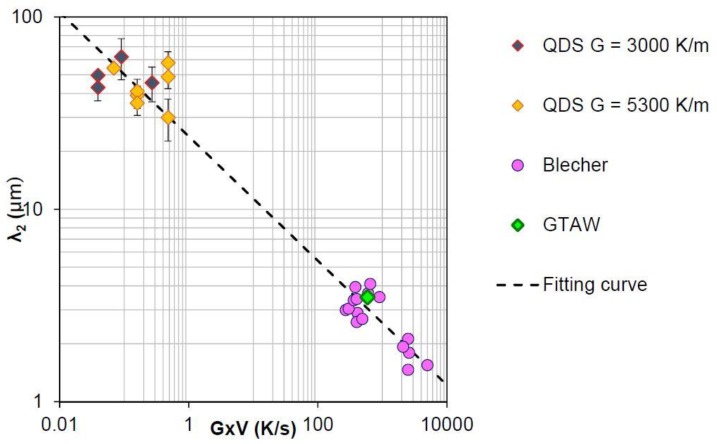
SDAS evolution of FM52 alloy as function of the cooling rate in K/s for QDS, GTAW experiments and Blecher et al.’s [45] measurements.

This data was supplemented by the measurements of Blecher et al. [45] in an A690 alloy, which had a composition very similar to the FM52 alloy. The SDAS evolution, with respect to the cooling rate, is plotted in Figure 2, using the Frenk and Kurz method [46]. The equation of the fitted curve, presented as a dashed line in Figure 2, is given by Equation (1). This law is very similar to the Blecher et al. law [45], and is very close to the well-known SDAS law for metallic alloys, $\lambda_{2}=A\times {\left(G\times V\right)}^{\frac{1}{3}}$, where A is a material constant [47].

(1) $$\lambda_{2}=23.92\times {\left(G\times V\right)}^{-0.323}\;\mathrm{in}\;\mathrm{microns}.$$

### 3.2. Microsegregation Results

**Figure 3:**
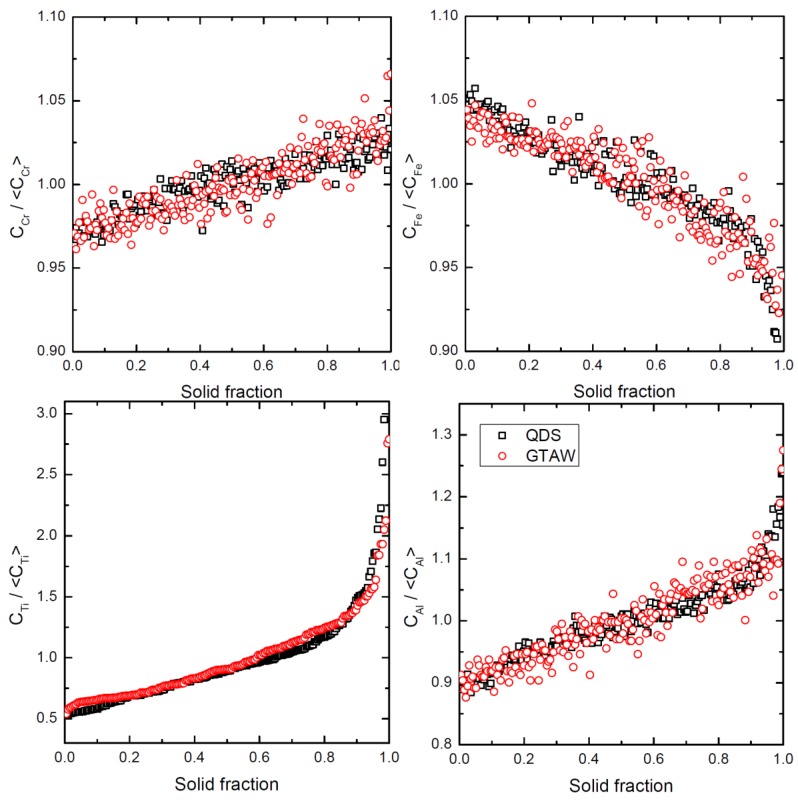
Comparison of microsegregation profiles between QDS, GV = 0.477 K/s and GTAW experiments.

The microsegregation profiles for the QDS and GTAW experiments are given for Fe, Cr, Ti and Al in Figure 3. Even though the cooling rate was a thousand times larger in QDS than GTAW, the microsegregation profiles do not differ significantly. In fact, they are identical for all elements (Figure 3), despite the cooling rates differing by three orders of magnitude.

The trends in the segregations shown in Figure 3 are comparable to others reported for different nickel alloys containing chromium, titanium and aluminum [48,49]. These elements segregate in the liquid during solidification, as opposed to iron which does not.

Two methods of estimating the partition coefficient were considered. From the phase diagram, the partition coefficient, denoted *k*_1_ in the following, was estimated using Equation (2). QDS offers a second possibility of estimating the partition coefficient if the microsegregation is analyzed on a transverse section, where the solid fraction is less than 1. This method was used to calculate the partition coefficient, denoted *k*_2_, defined in Equation (3). The second method was interesting as it was able to evaluate the influence of the composition at the interface and the temperature on the partition coefficient.

Due to the small solidification range of the FM52 alloy, the length of the mushy zone was only ~2 mm. It was therefore difficult to accurately cut. Nevertheless, it was successfully sectioned on the QDS test for GV = 0.039 K/s. In the analyzed region, the solid fraction was estimated to be approximately 80%.

(2) $$k_{1}=\frac{\mathrm{Solid}\;\mathrm{composition}\;\mathrm{for}\;f_{s}=0}{\mathrm{Mean}\;\mathrm{composition}}$$

(3) $$k_{2}=\frac{\mathrm{Solid}\;\mathrm{composition}\;\mathrm{at}\;\mathrm{quench}\;\mathrm{moment}}{\mathrm{Mean}\;\mathrm{composition}\;\mathrm{of}\;\mathrm{quenched}\;\mathrm{liquid}}$$

The values of the partition coefficients are presented in Table 4. Over three cooling rates and two solid fractions, the values did not have significant variation. This indicates that the partition coefficients do not depend on the composition and temperature changes at the interface.

The experimental analysis was concluded with partition coefficients determined for alloy FM52 and the ternary alloy approximation from the thermodynamic TTNi8 database. The small effect of composition on the majority element partition coefficients should be noted. The data for the ternary and FM52 alloys were similar. The values presented in Table 4 differ somewhat between the measured and calculated. In the Thermo-Calc computation, the undercooling was not considered, so the first solid that formed had the same composition as was predicted by the phase diagram, i.e., the product between the nominal composition and the partition coefficient. These results supported the assumption that the FM52 alloy behaved like the considered ternary alloy, as was assumed for the following model. 

In addition to the thermodynamic data, the most surprising result was that the microsegregation profiles were identical in QDS and GTAW, which could not be explained by a size effect (Fourier number was of the order of 0.3 in QDS, compared to 0.025 in GTAW). Several factors had to be considered in order to explain this. First, it was necessary to clarify the role of convection (important in welding) on undercooling, and by extension on microsegregation. Next, attention had to be paid to the size of the structures and to the experimental determination of the microsegregation. These two questions were not considered by Liang and Chen [50].

## 4. Modelling Microsegregation: Including Fluid Flow

### 4.1. Microsegregation Model

The most common microsegregation models are the well-known lever rule and Scheil model [51]. The lever rule assumes an infinite diffusion in both the liquid and the solid phases, while the Scheil model assumes no diffusion in the solid phase and infinite diffusion in the liquid phase. They are usually used to evaluate the possible microsegregation range, but cannot be considered fully realistic. Many more elaborate microsegregation models have been proposed over the years, most based on the Clyne–Kurz (CK) model [52], the Kobayashi model [53], the Wang–Beckermann model [54] or the Tong–Beckermann (TB) model [35]. In the present work, only the TB model was considered. 

Compared to the Scheil model, the TB model accounts for solute diffusion in the solid phase, through a solutal Fourier number, *α*, defined in the CK model as [52]:

(4) $$\alpha_{i}=\frac{D_{i}^{s}t_{f}}{\lambda_{2}^{2}}$$

where $D_{i}^{s}$ is the diffusion coefficeient for the solute *i* in the solid phase, *t_f_* is the local solidification time, defined as $\Delta T_{0}/G_{T}V$, and *λ*_2_ is the secondary dendrite arm spacing. *α* is also known as the back-diffusion parameter. 

In addition to the CK model, the TB model considers limited diffusion both into the solid and liquid phase, which can be relevant when the solidification velocity is important, such as is the case in welding operations. Similar to the CK model, the TB model introduces a solutal Fourier number *β* for diffusion in the liquid phase:

(5) $$\beta =\frac{D_{i}^{l}t_{f}}{\lambda_{2}^{2}}$$

where $D_{i}^{l}$ is the solute diffusion coefficient in the liquid phase. This Fourier number may also be used to define a second adimensional parameter called *β*’, introducing a “tuning constant”, *σ*, defined as:

(6) $$\beta^{\prime }=\sigma \beta .$$

This constant is included in the model to compensate for the error introduced by the solute diffusion of the moving solid/liquid interface, which does not satisfy the zero flux condition at the symmetry line separating two dendrites [35]. In practical terms, this tuning constant accounts for the dendrite tip undercooling and changes the composition of the first solid, accordingly. The microsegregation evolution during solidification can be obtained by solving the following differential equation for solid fraction *f_s_* ranging from 0 to 1:

(7) $$\frac{2\beta^{\prime }f_{s}}{k_{i}}\left(1-\delta \right)\frac{dw_{i}^{s*}}{df_{s}}=\left(1+6\alpha \right)\left(w_{i}^{0}-w_{i}^{s*}\right)+\left(\frac{w_{i}^{s*}}{k_{i}}-w_{i}^{0}\right)\left(\frac{\delta }{f_{s}}-2\beta^{\prime }\left(1+6\alpha \right)\left(1-\delta \right)\right)$$

where $w_{i}^{0}$ is the nominal mass fraction, $w_{i}^{s*}$ is the interfacial solid mass fraction at a given *f_s_*, $k_{i}$ is the partition coefficient for the solute *i*, and:

(8) $$\delta =\exp\left(-\frac{1-f_{s}}{2\beta^{\prime }f_{s}}\right).$$

Note that Equation (4) has corrected a typo found in Equation (14), published in Reference [35]. In order to solve Equation (7), the initial solid composition $w_{i}^{s}\left(f_{s}\to 0\right)$ must be determined. This initial solid composition actually corresponds to the dendrite tip composition.

### 4.2. Tip Undercooling 

To establish the dendrite tip composition, the tip operating point must be determined by ascertaining the dendrite tip undercooling:

(9) $$\Delta T=\Delta T_{T}+\Delta T_{R}+\Delta T_{C}+\Delta T_{k}$$

where $\Delta T_{T}$ is the thermal undercooling, $\Delta T_{R}$ is the curvature undercooling, $\Delta T_{C}$ is the chemical or solutal undercooling, and $\Delta T_{k}$ the kinetic undercooling. In the processing conditions considered in this paper, $\Delta T_{T}$ and $\Delta T_{k}$ were neglected. The curvature undercooling is equal to Γ/*r* where Γ is the Gibbs–Thomson coefficient and *r* is the dendrite tip radius. The determination of the chemical undercooling can be achieved using the well-known KGT model [33]. In this work, to account for fluid flow that can be significant in the GTAW process, a modified formulation of the KGT model derived by Appolaire et al. was chosen [34]. The KGT model considers an isolated dendrite with a tip described by a circular paraboloid. At the interface, the liquid composition $w_{l}^{*}$ is assumed to be homogeneous along the surface of the paraboloid, and at infinity from the nominal solute composition *w*_0_. Accounting for fluid flow, the supersaturation for each solute *i* $\Omega_{i}=\left(w_{i}^{l*}-w_{i}^{0}\right)/\left(w_{i}^{l*}\left(1-k_{i}\right)\right)$ is related to two Péclet numbers: (i) the solutal Péclet number $P_{i}=rV/2D_{i}^{l}$ and the flow Péclet number $P_{u_{i}}=rU/2D_{i}^{l}$, where U the relative velocity between solid and liquid:

(10) $$\Omega_{i}=F\left(P_{i},P_{u_{i}}\right).$$

For the range of tip radii and fluid flow velocities encountered in this work, the Stokes regime was considered appropriate [55], and thus function F could be given by:

(11) $$F\left(P_{i},P_{u_{i}}\right)=2P_{i}\int_{1}^{\infty }\exp\left\{-\ln\eta +\left(1-\eta^{2}\right)P_{i}-2P_{u_{i}}/E_{1}\left(Re_{t}\right)\left[1-\eta^{2}+\left(1-\eta^{2}\right)\ln\eta \right]\;-2\varepsilon P_{i}\ln\eta \right\}d\eta$$

where *η* is the coordinate in the paraboloid frame. The surface of the tip is located at *η* = 1. $Re_{t}=\left(P_{i}+P_{u_{i}}\right)/Sc_{i}$; $Sc_{i}$ is the Schmidt number of the solute *i*, found by the equation $Sc_{i}=\upsilon /D_{i}^{l}$, where *ν* is the kinematic viscosity of the liquid; and *ε* is the relative density difference between the solid and liquid phases, $\varepsilon =(\rho_{s}/\rho_{l})-1$. This term plays an insignificant role on the supersaturation, and so it was neglected here. The chemical undercooling is then related to the interfacial mass fractions:

(12) $$\Delta T_{C}=m\left(w_{i}^{0}-w_{i}^{l*}\right)$$

where *m* is the liquidus slope of the element. The final expression for the total undercooling becomes:

(13) $$\Delta T=\frac{\Gamma }{r}+\underset{i}{\sum }w_{i}^{0}m_{i}\left[1-\frac{1}{1-\left(1-k_{i}\right)F\left(P_{i},P_{u_{i}}\right)}\right]$$

and the associated value of $w_{i}^{s}\left(f_{s}\to 0\right)$ is:

(14) $$w_{i}^{s}\left(f_{s}\to 0\right)=\frac{k_{i}w_{i}^{0}}{1-\left(1-k_{i}\right)F\left(P_{i},P_{u_{i}}\right)}$$

(15) $$w_{i}^{s}\left(f_{s}\to 0\right)=\frac{1+2\beta^{\prime }}{1+\frac{2\beta^{\prime }}{k}}\times w_{i}^{0}.$$

By coupling Equation (15) from Reference [35] with Equation (11) from this work, the following expression of *σ* was obtained for each solute *i*:

(16) $$\sigma_{i}=\frac{1}{\beta }\left(\frac{1-F\left(P_{i},P_{u_{i}}\right)}{2F\left(P_{i},P_{u_{i}}\right)}\right)$$

The obtained value for $\sigma_{i}$ was then used to determine $\beta^{\prime }$ and thus Equation (7) was solved, giving the evolution of the solute mass fraction for solid fractions ranging from 0 to 1. 

### 4.3. Consequence of Convection on Microsegregation

Gabathuler and Weinberg [56] showed that fluid flow does not penetrate the dendritic skeleton above $f_{s}>0.2$, while Appolaire et al. [55] showed that fluid flow led to a faster increase of the solid fraction at low solid fractions. At higher solid fractions, the flow is assumed to be described by Darcy’s equation, which relates the flow velocity to the pressure gradient in the mushy zone. Defining the local convection time in the mushy zone as $t_{conv}=2\lambda_{2}/U_{MZ}$, where $U_{MZ}$ is the flow velocity in the mushy zone, it can be compared to the local solidification time, $t_{f}$, to determine whether or not the flow has an impact on microsegregation. In the present work $t_{f}\ll t_{conv}$ for all the investigated processing conditions. It should be noted that this would be different in the case where cellular solidification occurs, as this would involve a change in the characteristic length from the secondary dendrite arm spacing to the primary dendrite arm spacing, and a sharp increase in the permeability. In this case, it is assumed that convection will not affect the microsegregation, except for the initial solid composition.

### 4.4. Evaluation of Tip Undercooling in the Presence of Convection

Table 5 shows the data used for the calculations, wherein a ternary Ni-Cr-Fe alloy was considered and the liquidus and solidus planes were linearized [33]. The Gibbs–Thompson coefficient was approximated with the equation of Magnin and Trivedi [57]. The diffusion coefficients in the liquid were evaluated using the Stokes–Einstein relation. The dynamic viscosity was given by Reference [58]. The Thermo-Calc software, in conjunction with the TTNi8 database, were used to determine the phase equilibrium data. The latent heat of fusion was obtained experimentally by DTA. The value we obtained was close to that used in Reference [40]. 

**Figure 4:**
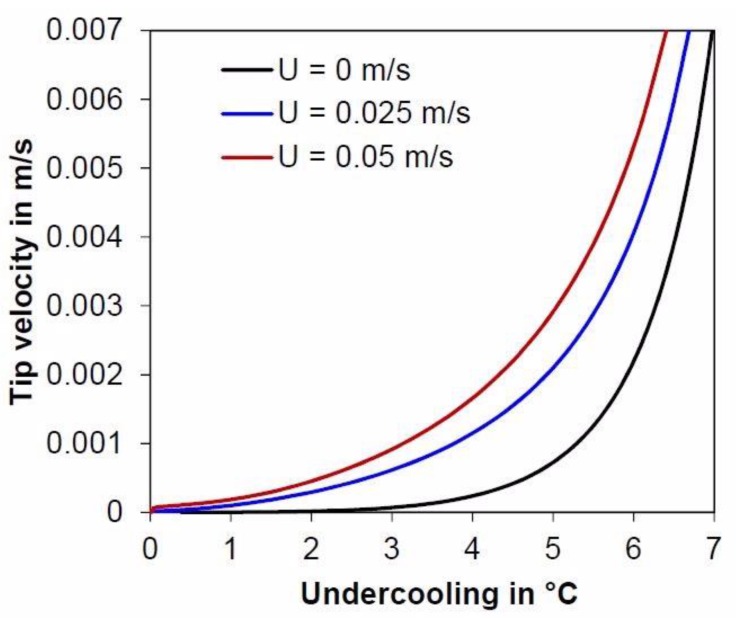
Undercooling as a function of the tip velocity for several fluid flow intensity convections.

Figure 4 shows the undercooling evolution as a function of the tip velocity for different liquid convections. The black line is the classical KGT computation. The blue and red lines are computations with convection in the melt for flow velocities of 0.025 and 0.05 m/s, respectively. These speeds are of the typical order of magnitude for convection at the bottom of the molten bath in GTAW, and can reach several tens of centimeters per second at the surface of the bath [32]. The analyzed region for microsegregation in GTAW is located at the bath bottom, so 0.025 m/s was the value used. For QDS, the convection was considered equal to zero. As can be seen in Figure 4, convection reduces the tip undercooling in GTAW nearer to values encountered in QDS. 

## 5. Comparison between Microsegregation Model and Experimental Results

### 5.1. QDS Experiment

**Figure 5:**
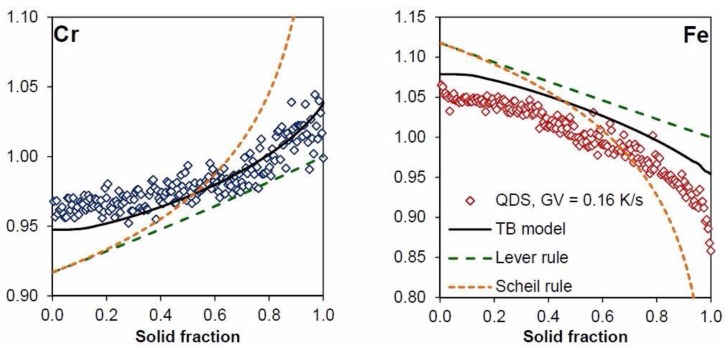
Comparison between microsegregation profile and Tong–Beckermann (TB) model for QDS experience for chromium and iron for GV = 0.16 K/s.

Figure 5 shows the different microsegregation profiles for one QDS experiment, along with the microsegregation predicted by the TB model, lever rule and Scheil rule. 

The model was able to predict the observed trend at low solid fractions (between 0 and 0.2) in the case of iron, as shown in Figure 5. The trend was less pronounced for chromium, but still present. The importance of taking into account the undercooling, in particular for the small solid fractions (up to 0.3 fs) on the micro-segregation, may be recognized from this data.

The experimental data points and the TB model are slightly shifted apart from one another, in particular for the first solid formed. This could be due to the fact that the TB model computes the composition of solid at the interface with homogenization during the local solidification time. Post homogenization, occurring during subsequent cooling at the dendrite scale, was not taken into account by the model, although it does occur in the experiment. Simplification when computing the data, by assuming the alloy FM52 to be a ternary alloy, was also carried out.

Nevertheless, the curves given by the TB model compare well with the experimental data despite the slight shift previously noted. It should be remembered that the difference between the compositions of the first solid—detected by EPMA and calculated by the TB model—was small. This deviation corresponds to 0.45% and 0.25% by mass, in the case of chromium and iron, respectively. The TB model, coupled with the undercooling calculation, was then validated by the experiments in order to predict the microsegregation profiles. 

### 5.2. TB Model and GTAW

**Figure 6:**
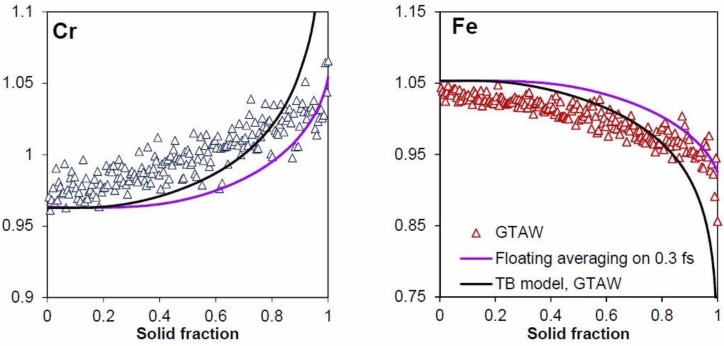
Comparison between the microsegregation profiles given by the TB model, and the TB model with floating averaging in the case of the GTAW experiments.

Figure 6 shows the Cr and Fe microsegregation profiles for the GTAW experiments, and the microsegregation predicted by the TB model. The Lever and Scheil rule are not shown for the sake of clarity. A good fit between the model and the experiments can be observed, except at high solid fractions. However, the effect of microstructure size should also be considered, not only in the modeling parameters—which take into account back-diffusion and finite liquid diffusion—but also in the experiments, because of the fineness of the microstructures. The EPMA probe smoothed the composition gradient due to the interaction volume of the electrons with the analyzed material. In the case of welding, the interdendritic spacing investigated by the microprobe was SDAS. Following the Castaing relation [62] to determine the depth of penetration of the electron beam, using a classical voltage of 20 kV, a 0.850 µm depth interaction zone was predicted. Generally, 1 micron is often used. Thus, the solid fraction analyzed at each microprobe pointed could be approximated by a $\frac{\mathrm{depth}\;\mathrm{penetration}}{\mathrm{SDAS}}$ ratio. 

In the case of welding, the SDAS was approximately 3 or 4 microns. Thus, the primary phase solid fraction over this distance must vary from 0 to 1. If the diameter of the microprobe interaction volume is one micron, each analysis point accounts for one third or one quarter of the solid fraction. This must be taken into consideration when comparing the predicted microsegregation profiles to the experimental. In order to do so in this study, a floating average method was used on the model output concentration, ranging from +/−15% solid fraction, for each output point. The results of this method are also plotted in Figure 6. 

With the floating average, the TB curves correspond relatively well to the experimental measurements, especially in the case of iron. The slight difference between the microprobe points and the floating averaging TB model is likely caused by the use of the magnitude approximation for the convection in the molten pool.

## 6. Conclusions

The microsegregation behaviors and undercooling of the alloy FM52 were investigated using quenched directional solidification and gas tungsten arc welding. The partition coefficients were confirmed to be close to unity for most alloy elements. Microsegregation was experimentally characterized. Despite the large range of solidification conditions used, it was found that microsegregation does not change drastically. The Tong–Beckerman microsegregation model was then modified to take into account the effect of fluid flow on dendrite tip undercooling. With such modification, the TB model fits well with the experimental data, especially when the interaction volume of the experimental analysis was considered. This modified TB model can be used for microsegregation predictions in all solidification conditions, which, when compared with other analytical models, improves the precision without drastically increasing the complexity.

## Figures and Tables

**Table 1:** Composition of alloy filler metal 52 (FM52) in weight percent.

	Ni	Cr	Fe	Al	Ti	Mn	Si	C
EPMA	57.98	29.98	10.06	0.65	0.52	0.29	0.13	0.03
Specification [36]	≈60	28–31.5	7–11	<1.1	<1	<0.5	<0.5	<0.04

**Table 2:** Results of the differential thermal analyses (DTA) and thermodynamic calculations with the TTNi8 database.

	DTA (°C)	Thermo-Calc (°C, FM52)	Thermo-Calc (°C, Ni-29.98Cr-10.06Fe)
Solidus	1379.2	1364	1409.4
Liquidus	1389.8	1400	1417.8

**Table 3:** Solidification parameters used in the quenched directional solidification (QDS) and welding experiments.

Test Type	Thermal Gradient (K/m)	*V* (mm/s)	*G*/*V* (K·s/m²)	*G*·*V* (K/s)
QDS	5300	0.09	5.89 × 10^7^	0.477
		0.03	1.77 × 10^8^	0.159
		0.013	4.08 × 10^8^	0.0689
	3000	0.09	3.33 × 10^7^	0.27
		0.03	1 × 10^8^	0.09
		0.013	2.31 × 10^8^	0.039
GTAW	≈300,000	1.66	1.81 × 10^8^	498

**Table 4:** Partition coefficients QDS, GTAW, and Thermo-Calc calculations.

	*k*	Cr	Fe	Al	Ti	Mn	Si
QDS, GV = 0.477 K/s	*k*_1_	0.96	1.05	0.87	0.5	0.75	0.59
QDS, GV = 0.16 K/s	*k*_1_	0.96	1.07	0.86	0.46	0.73	0.66
QDS, GV = 0.039 K/s	*k*_1_	0.98	1.06	0.87	0.5	0.76	0.66
	*k*_2_	0.98	1.05	0.94	0.62	0.71	0.59
GTAW	*k*_1_	0.97	1.04	0.91	0.54	x	x
Thermo-Calc for FM52 alloy	*k*_1_	0.93	1.13	0.99	0.41	0.54	0.62
Thermo-Calc for ternary Ni-29.98Cr-10.06Fe	*k*_1_	0.92	1.11	x	x	x	x

**Table 5:** Physical and thermodynamic data used for the calculations.

**Physical Quantities**	
Gibbs-Thompson coefficient (Km)	Γ = 1.82 × 10^−7^
Diffusion Coefficient for Fe and Cr in molten nickel (m^2^/s)	D_l_ = 1.57 × 10^−9^
Heat capacity of liquid (J·kg^−1^·K^−1^) [59,60]	Cp_l_ = 700
Thermal conductivity of liquid (W·m^−1^·K^−1^) [59,61]	λ_l_ = 30
Dynamic viscosity of liquid at 1400 °C (N·s·m^−2^) [58]	μ = 0.00483
Liquid density at T_l_ (kg·m^−3^) [58]	ρ_l_ = 7160
Latent heat of fusion (J/kg)	L = 1.3 × 10^5^
**Thermodynamic Quantities**	
Liquidus slope for Cr (K·(%w)^−1^)	m_Cr_ = −2.27
Liquidus slope for Fe (K·(%w)^−1^)	m_Fe_ = 4.55
Partition coefficient of Cr	k_Cr_ = 0.93
Partition coefficient of Fe	k_Fe_ = 1.11
Composition of Cr in weight percent	29.98
Composition of Fe in weight percent	10.06
Fictive reference temperature in °C [33]	1439.5
Solidus temperature	1409.4
Liquidus temperature	1417.8
